# Emergence of Erythromycin-Resistant Invasive Group A *Streptococcus*, West Virginia, USA, 2020–2021

**DOI:** 10.3201/eid2905.221421

**Published:** 2023-05

**Authors:** Lillie M. Powell, Soo Jeon Choi, Chloe E. Chipman, Megan E. Grund, P. Rocco LaSala, Slawomir Lukomski

**Affiliations:** West Virginia University, Morgantown, West Virginia, USA

**Keywords:** invasive group A Streptococcus, antimicrobial resistance, streptococci, Streptococcus pyogenes, erythromycin, clindamycin, West Virginia, emm92, inducible, constitutive, drug resistance, bacteria, United States

## Abstract

Clindamycin and β-lactam antibiotics have been mainstays for treating invasive group A *Streptococcus* (iGAS) infection, yet such regimens might be limited for strains displaying MLS_B_ phenotypes. We investigated 76 iGAS isolates from 66 patients in West Virginia, USA, during 2020–2021. We performed *emm* typing using Centers for Disease Control and Prevention guidelines and assessed resistance both genotypically and phenotypically. Median patient age was 42 (range 23–86) years. We found 76% of isolates were simultaneously resistant to erythromycin and clindamycin, including all *emm92* and *emm11* isolates. Macrolide resistance was conferred by the plasmid-borne *ermT* gene in all *emm92* isolates and by chromosomally encoded *ermA*, *ermB*, and a single *mefA* in other *emm* types. Macrolide-resistant iGAS isolates were typically resistant to tetracycline and aminoglycosides. Vulnerability to infection was associated with socioeconomic status. Our results show a predominance of macrolide-resistant isolates and a shift in *emm* type distribution compared with historical reports.

*Streptococcus pyogenes,* also known as group A *Streptococcus* (GAS), is a ubiquitous pathogen that produces an array of human disease, including focal infections (e.g., pharyngitis, pyoderma) with or without localized suppurative complications; invasive soft tissue infections (e.g., myositis, necrotizing fasciitis); and systemic, often fatal, infections (e.g., bacteremia, toxic shock syndrome). In addition, 2 postinfectious complications (glomerulonephritis and rheumatic heart disease) attributable to GAS have been well described (*1*–*3*). Although GAS remains susceptible to penicillin, treatment with alternative or combination therapies, such as macrolides, clindamycin, and other second-line antimicrobial medications, is common because of patient β-lactam allergies, dosing convenience, infection severity, and patient acuity (*4*). In contrast to its predictable β-lactam susceptibility, GAS resistance to other classes of antimicrobial drugs has been increasingly reported (*5*–*7*). In the face of ongoing dissemination of the MLS_B_ (macrolide, lincosamide, and streptogramin B) resistance phenotypes among GAS isolates, the Centers for Disease Control and Prevention (CDC) has labeled macrolide-resistant GAS an emerging threat of concern (*8*).

As 1 component of its Active Bacterial Core surveillance (ABCs) system, the CDC Emerging Infections Program, part of the National Center for Emerging and Zoonotic Infectious Diseases, Division of Preparedness and Emerging Infections, provides ongoing population-based assessments of GAS infections from 10 sites in the United States. Annual reports produced by the program estimate the incidence of invasive GAS (iGAS) infections within the United States doubled from 2009 to 2019; total numbers of infections increased from ≈11,000 cases (3.6 cases/100,000 population) to >25,000 cases (7.6 cases/100,000 population) (*9*,*10*). Concomitant with this change, substantial increases in the proportion of iGAS isolates resistant to erythromycin and clindamycin have been reported; overall resistance rates climbed from <10% in 2010 to near 25% by 2017 (*11*). Populations at risk for such macrolide-resistant iGAS infections have been predominantly persons 18–64 years of age; incidence is high among persons with a history of intravenous drug use (IVDU) and persons experiencing homelessness (*11*,*12*).

West Virginia, USA, has seen a noticeable increase in annual rates of iGAS erythromycin resistance; at West Virginia University Medicine System (WVUMed) hospitals in Morgantown, rates increased from 37% in 2019 to 54% in 2020 and 87% in 2021. The state also has an extremely high per capita rate of drug overdose (*13*). On the basis of all those considerations, we conducted a study to review clinicoepidemiology of iGAS infections within the region and to characterize specific phenotypic and genotypic antimicrobial resistance traits of corresponding available isolates.

## Materials And Methods

### Study Setting

The clinical laboratory at J.W. Ruby Memorial Hospital in Morgantown serves as the primary reference facility for all 19 WVUMed hospitals located throughout West Virginia, as well as facilities in western Maryland, southwestern Pennsylvania, and eastern Ohio. The WVUMed system serves an estimated patient population of 1.2 million. Most microbiological testing at J.W. Ruby Memorial Hospital and surrounding WVUMed outpatient clinics is performed by the Ruby clinical laboratory, as is referral antimicrobial susceptibility testing of many *Streptococcus* spp. isolates. The clinical laboratory routinely banks invasive isolates at −80^ᵒ^C for 1–2 years. Noninvasive isolates, including those recovered from pharyngitis cases, are not routinely held >7 days after specimen submission.

The strain collection for this study included all viable primary and referred iGAS isolates available from the freezer bank, which spanned the period January 2020–June 2021. After approval by the hospital’s Institutional Review Board (protocol no. 2202533507), we reviewed patient records to capture demographic and clinical information, such as patient age, sex, residence status, history of IVDU, intensive care unit admission requirement, number of surgical interventions, antimicrobial regimen, and clinical outcome (Appendix Table 1), for all isolates successfully retrieved. We included 2–4 replicate isolates recovered serially from 8 patients and tested them separately as a quality control measure. In all instances, intrapatient phenotypic and genotypic results were consistent, so isolates are reported per patient throughout.

### Chromosomal and Plasmid DNA Isolation

We isolated genomic DNA from a 10-μL loopful of bacteria grown in Todd Hewitt broth by using the DNA extraction procedure, as described previously (*14*). We isolated plasmid DNA by using Gene JET Plasmid Miniprep Kit (ThermoFisher Scientific) with an additional cell-digestion step (1 mg/mL lysozyme and 0.5 U/µL mutanolysin) at 37°C for 1 hour. We analyzed plasmid DNA, uncut and digested with *Swa*I, on a 0.8% agarose gel for confirmation of plasmid pRW35 size in *emm92* isolates (*15*).

### Identification of Resistance Genes *erm/mef* and *emm* Typing

We used plasmid DNA as a PCR template with the *ermT*-specific primers, whereas we used genomic DNA as the PCR template with primers detecting *ermA(TR)*, *ermB(AM)*, and *mefA* genes (Appendix Table 2). We obtained control GAS strains harboring the corresponding *erm* and *mef* genes from the CDC *Streptococcus* Laboratory (https://www.cdc.gov/streplab/index.html). We used genomic DNA to determine isolate *emm* type by Sanger sequencing of amplicons generated with primers *emm*1b and *emm*2 (*16*), followed by BLAST search (https://blast.ncbi.nlm.nih.gov/Blast.cgi) on the CDC *Streptococcus* Laboratory.

### Antimicrobial Susceptibility Testing

We performed erythromycin, clindamycin, and tetracycline susceptibility testing in the clinical microbiology laboratory by using methods described by the Clinical Laboratory Standards Institute (CLSI) (*17*). All automated testing used Vitek 2 ST-02 cards (bioMériuex) and was performed upon isolate recovery for clinical management purposes. All valid results were reported to and retrieved from patient electronic health records (Epic). All historic testing met ongoing quality control criteria as outlined in the laboratory Quality Management Plan and Individualized Quality Control Plan as required for laboratory accreditation. Subsequently, we performed disc diffusion and D-testing over multiple days using thawed isolates from the freezer bank. After 2 serial propagations on sheep blood agar, we inoculated swabs of 0.5 MacFarland suspensions for confluent growth on cation-adjusted Mueller Hinton agar with 5% sheep blood (BD) using discs containing conventional drug masses. Quality control organisms, including ATCC BAA-977, ATCC BAA-976, and ATCC 49619, were tested in parallel each day of use. We incubated plates at 35^ᵒ^C in 5% CO_2_ environment for 20–24 h before measuring zones of inhibition with a manual caliper in reflected light. We interpreted zone diameters by using CLSI clinical breakpoints (*17*) and interpreted any degree of clindamycin zone flattening in proximity to erythromycin disc as a positive D-test result.

Susceptibility testing against aminoglycosides (gentamicin, kanamycin, and streptomycin) was performed by agar dilution on Mueller Hinton media (BD) prepared in the research laboratory. A saline suspension of each isolate at an absorbance of 1 Klett unit was prepared and a 10-µL drop (≈10^4^ CFU) was plated in singlicate onto agar medium containing arbitrarily selected concentrations ranging from 50 to 500 μg/mL, as described (*5*). Plates were incubated at 37°C in 5% CO_2_ overnight, followed by observation of growth results.

## Results

### Patients

**Figure 1:**
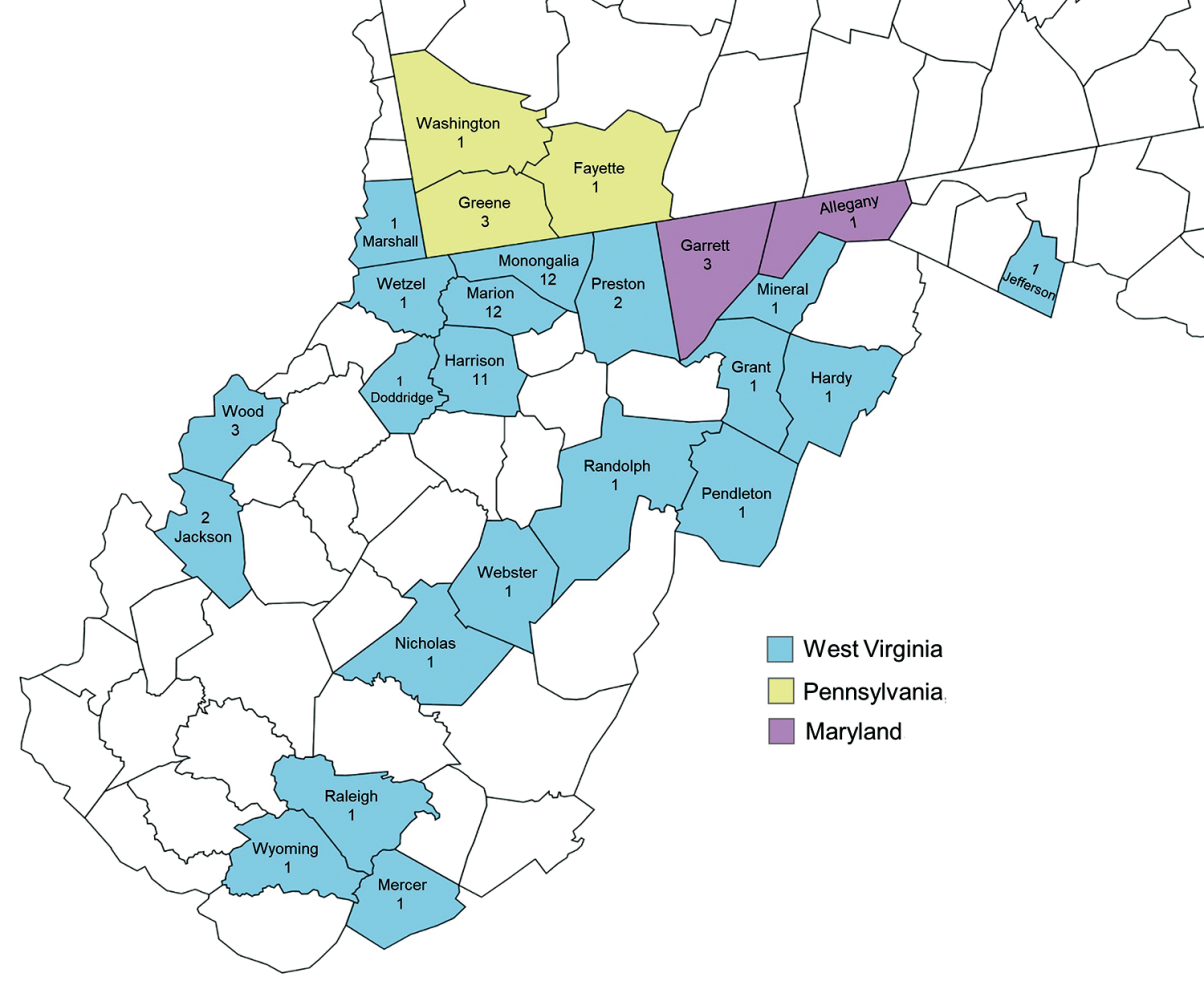
Geographic distribution of patients with invasive group A *Streptococcus* infection in West Virginia, USA, 2020–2021. A total of 56 iGAS isolates were collected from patients in 20 counties. Residence status for 9 patients was undocumented, and 9 patients were listed as homeless; in those cases, we used the county of residence for the billing address. Nine isolates were from neighboring counties in Pennsylvania and Maryland. The predominant *emm* type in the 3 West Virginia counties containing the most isolates was *emm92* (10 isolates in Harrison County, 6 in Marion County, and 8 in Monongalia County) (Appendix Table 3).

We included 76 GAS isolates collected during January 2020–June 2021 from 66 patients with invasive infections (Figure 1; Appendix Table 1). Median patient age was 42 (mean 45, range 23–86) years; 59% were men. On the basis of addresses listed in medical records, geographic distribution of all 56 in-state patients spanned 20 of the 55 West Virginia counties; 3 northern counties (Harrison, Marion, and Monongalia) accounted for the highest proportion (53%), likely because of larger populations and proximity to the main WVUMed campus in Morgantown. Most out-of-state patients were also within the WVUMed catchment area in neighboring counties in Maryland (n = 4) and Pennsylvania (n = 5) (Figure 1), although 1 patient was visiting from the Midwest United States. For 9 patients, details of housing status in their medical records were insufficient; among the remaining patients, 9 (16%) were reported by a case worker to be experiencing homelessness at the time of culture, despite having an address on file in their medical records. For 64 patients whose social history was sufficiently documented, 39 (61%) reported recent or remote IVDU.

**Figure 2:**
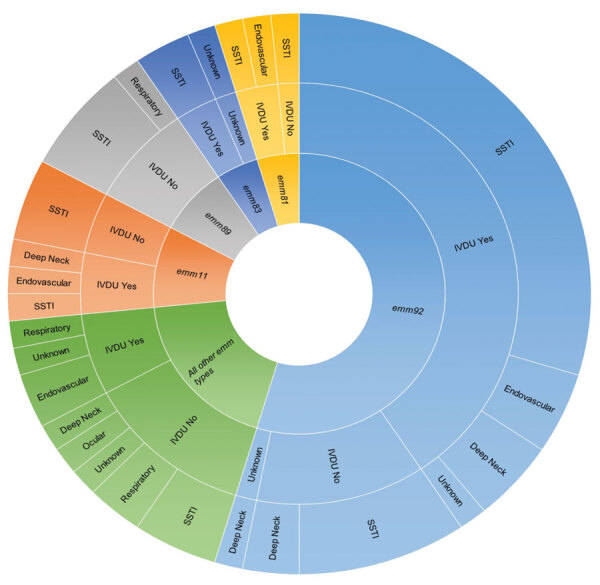
Assessment of *emm* type, infection source, and IVDU history of patients with invasive group A *Streptococcus* infection in this study, West Virginia, USA, 2020–2021. Anatomic source of infection and the status of patient IVDU history is shown corresponding to *emm* type. Size of the colored sections indicates the relative number of isolates per *emm* type. IVDU, intravenous drug use; SSTI, skin and soft tissue infection.

Assessing infection source for all 66 patients collectively revealed 38 (59%) with skin and soft tissue infections (SSTI) of the extremities: 8 (12%) with infections of deep neck structures; 6 (9%) with endovascular sources; 4 (6%) with SSTI of the gluteal, perianal, sacral, or inguinal region; 4 (6%) with respiratory sources; 1 (2%) with ocular source; and 4 (6%) with bloodstream infections of unknown origin (Figure 2). For an additional patient with a history of IVDU who had thrombophlebitis, bacteremia, and multiple SSTIs of the extremities and gluteal region, the primary source could not be discerned. A total of 35 (53%) patients required surgical intervention: single-stage debridement/washout (n = 24), fasciotomy with 2–13 serial debridements (n = 7), below-the-knee amputation revision (n = 1), tricuspid valve replacement (n = 1), thoracotomy with pleural decortication (n=1), and vascular thrombectomy (n = 1) (Appendix Table 1). Of the remaining nonsurgical patients, 8 underwent incision-drainage procedures or chest tube placement at bedside. Overall, 18 (27%) patients required admission to the intensive care unit for >24 hours and at least 5 (7.5%) died as a result of iGAS infection, although records of follow-up care were incomplete for a substantial portion of patients.

Antimicrobial therapy varied considerably by patient acuity, duration of hospitalization, intravenous catheter availability, and degree of initial treatment response. A total of 51 patients (77%) received >1 dose of intravenous vancomycin or daptomycin during hospitalization, whereas β-lactams (amoxicillin/clavulanate, cephems, penicillin, or a combination) or clindamycin were used exclusively for 10 patients (15%). Another 3 patients who were not admitted to the hospital received no antimicrobial therapy. In total, the acuity of infection for 9 patients did not require hospital admission. Another 10 patients left the hospital against medical advice before being admitted or completing treatment, 9 of whom had histories of IVDU. All these patients received prescriptions for oral clindamycin or amoxicillin/clavulanate at discharge. Among the remaining 47 patients, median hospital length of stay was 7 (mean 14, range 1–60) days.

### *emm* Types

**Figure 3:**
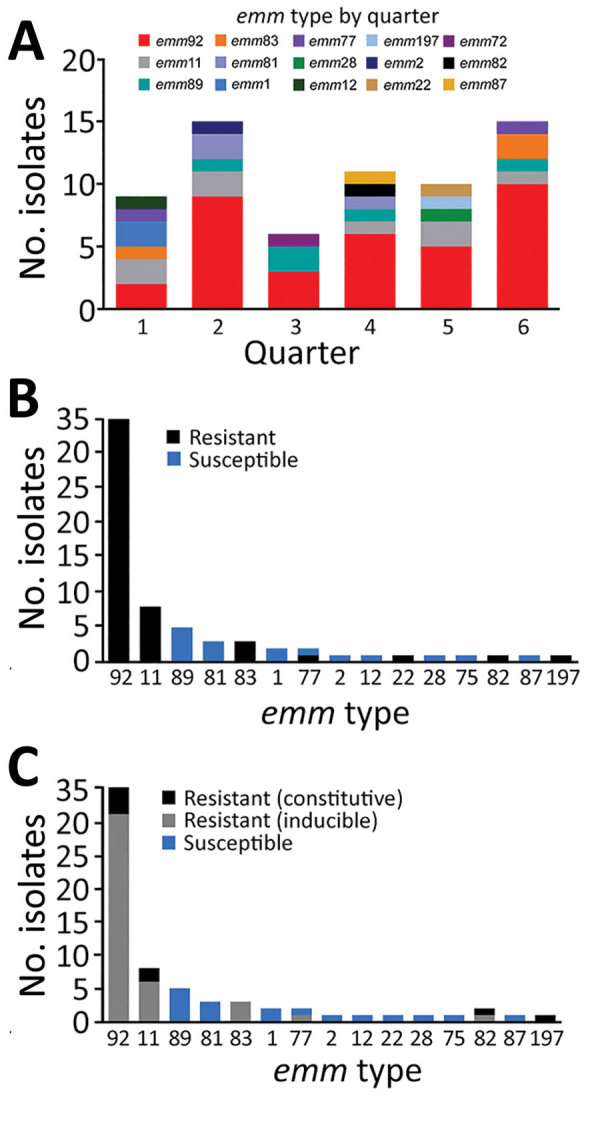
*emm* type distribution and MLS_B_ resistance among invasive group A *Streptococcus* isolates, West Virginia, USA, 2020–2021. A) Temporal analysis of isolate *emm* type by 3-month periods. Specimens harboring isolates were collected during January 2020–June 2021, represented by consecutive quarters numbered 1–6. Graph indicates trend of *emm92* isolates predominating each quarter over the study period. B, C) MLS_B_ susceptibility and resistance profiles. The number of isolates resistant to erythromycin (B) and clindamycin (C) by *emm* type was determined on the basis of antimicrobial susceptibility testing. Isolates were deemed nonsusceptible to clindamycin if they had either an inducible or constitutive resistance phenotype and deemed susceptible in the absence of growth as determined by D-test.

Although surveillance across the United States by ABCs has demonstrated an increase in iGAS, categorization of iGAS isolates in West Virginia was lacking. The M protein type of each isolate was determined by Sanger sequencing of the 5′ end of the *emm*-gene PCR product (Appendix Table 2), as described (*16*). Analysis showed the collection was predominated by isolates of 1 *emm* type; of the 66 unique patient isolates, 35 (53.0%) were *emm92* followed in decreasing proportion by *emm* types *emm11* (n = 8, 12.1%) and *emm89* (n = 5, 7.7%). (Table 1; Figure 3, panel A). Temporal analysis of isolate *emm* type recovery by 3-month periods showed an overall increase in isolates during April–June in 2020 and 2021; a substantial proportion of the isolates from all quarters were *emm* type 92. Although the presence of *emm11* and *emm89* isolates was relatively stable over time, the presence of *emm92* trended upward. Of note, the collection contained only 2 *emm1* isolates, 1 each of *emm12* and *emm28*, and no *emm3* isolates, which historically have been correlated with a high incidence of iGAS infections (Figure 3, panel A) (*1*,*18*,*19*). Although the data from this 1.5-year study period in West Virginia is less robust than ABCs national data, the findings do corroborate a continual shift in *emm* types responsible for iGAS disease, particularly among homeless populations and persons with a history of IVDU (*11*,*12*).

**Table 1:** Phenotypic antimicrobial susceptibility results in invasive group A *Streptococcus* isolates, by *emm* type and resistance determinant, West Virginia, USA, 2020–2021*

*emm* type	Geno, no.	No. (%)																	
		Erythromycin			Clindamycin				Tetracycline			Kanamycin			Streptomycin			Gentamicin	
		S	R		S	iMLS_B_	cMLS_B_		S	R		S	R		S	R		S	R
*emm92*	*ermT*, 35	0	35 (100)		0	31 (89)	4 (11)		0	35 (100)		0	35 (100)		0	35 (100)		35 (100)	0
*emm11*	*ermA*, 2	0	2 (100)		0	2 (100)	0		0	2 (100)		0	2 (100)		2 (100)	0		2 (100)	0
	*ermB*, 6	0	6 (100)		0	3 (50)	3 (50)			6 (100)		2 (33)	4 (67)		0	6 (100)		6 (100)	0
*emm77*	*ermA*, 1	0	1 (100)		0	1 (100)	0		0	1 (100)		0	1 (100)		1 (100)	0		1 (100)	0
	ND, 1	1 (100)	0		1 (100)	0	0		1 (100)	0		0	1 (100)		1 (100)	0		1 (100)	0
*emm83*	*ermA*, 3	0	3 (100)		0	3 (100)	0		0	3 (100)		0	3 (100)		2 (67)	1 (33)		3 (100)	0
*emm197*	*ermA*, 1	0	1 (100)		0	0	1 (100)		0	1 (100)		1 (100)	0		1 (100)	0		1 (100)	0
*emm82*	*ermB*, 1	0	1 (100)		0	0	1 (100)		0	1 (100)		1 (100)	0		1 (100)	0		1 (100)	0
*emm22*	*mefA*, 1	0	1 (100)		1 (100)	0	0		0	1 (100)		1 (100)	0		1 (100)	0		1 (100)	0
*emm89*	ND, 5	5 (100)	0		5 (100)	0	0		5 (100)	0		NT			NT			5 (100)	0
*emm*†	ND, 10	10 (100)	0		10 (100)	0	0		9 (90)	1 (10)		2 (20)	8 (80)		9 (90)	1 (10)		10 (100)	0
Total = 66		16 (24)	50 (76)		17 (25)	40 (61)	9 (14)		15 (23)	51 (77)		7 (11)	54 (89)		24 (39)	37 (61)		66 (100)	0

*Geno, genotype; ND, resistance gene not detected; NT, not tested; R, resistant; S, susceptible. †One *emm11* isolate was not inducible by D-test but showed intermediate clindamycin resistance; other *emm* types with no *erm: emm1* (2), *emm2* (1), *emm12* (1), *emm28* (1), *emm75* (1), *emm81* (3), *emm87* (1).

### MLS_B_ Susceptibility and Resistance Profiles

Next, we assessed antimicrobial resistance among isolates in our iGAS collection. In aggregate, 76% (50/66) of isolates were resistant to erythromycin (Table 1), which is considerably higher than the percentage reported in a larger collection (*11*). Aside from *emm77*, which included 1 erythromycin-resistant isolate and 1 erythromycin-susceptible isolate, all other *emm* types exclusively harbored either erythromycin-resistant or erythromycin-susceptible phenotypes (Table 1; Figure 3, panel B). We used disc diffusion and D-testing to assess clindamycin susceptibility and to determine whether resistance was constitutive or inducible (Figure 3, panel C). Clindamycin susceptibility mirrored that of erythromycin; 16 erythromycin-susceptible isolates also demonstrated clindamycin susceptibility. Of the 50 erythromycin-resistant isolates, 40 exhibited inducible clindamycin resistance (i.e., not detectable without erythromycin induction), 9 demonstrated constitutive clindamycin resistance, and 1 isolate (*emm*22) was clindamycin susceptible without evidence of inhibition zone flattening. Similar to erythromycin, most *emm* types were uniformly susceptible or resistant to clindamycin except for 2 *emm*77 (1 susceptible and 1 inducible-resistant isolate) (Table 1; Figure 3, panel C). Phenotypic heterogeneity was also noted among *emm*92 isolates (of which 4 exhibited constitutive clindamycin resistance and 31 exhibited inducible clindamycin resistance), as well as among *emm*11 isolates (of which 5 isolates produced inducible and 3 produced constitutive phenotypes) (Table 1).

### Detection of Erythromycin-Resistance Determinants

**Figure 4:**
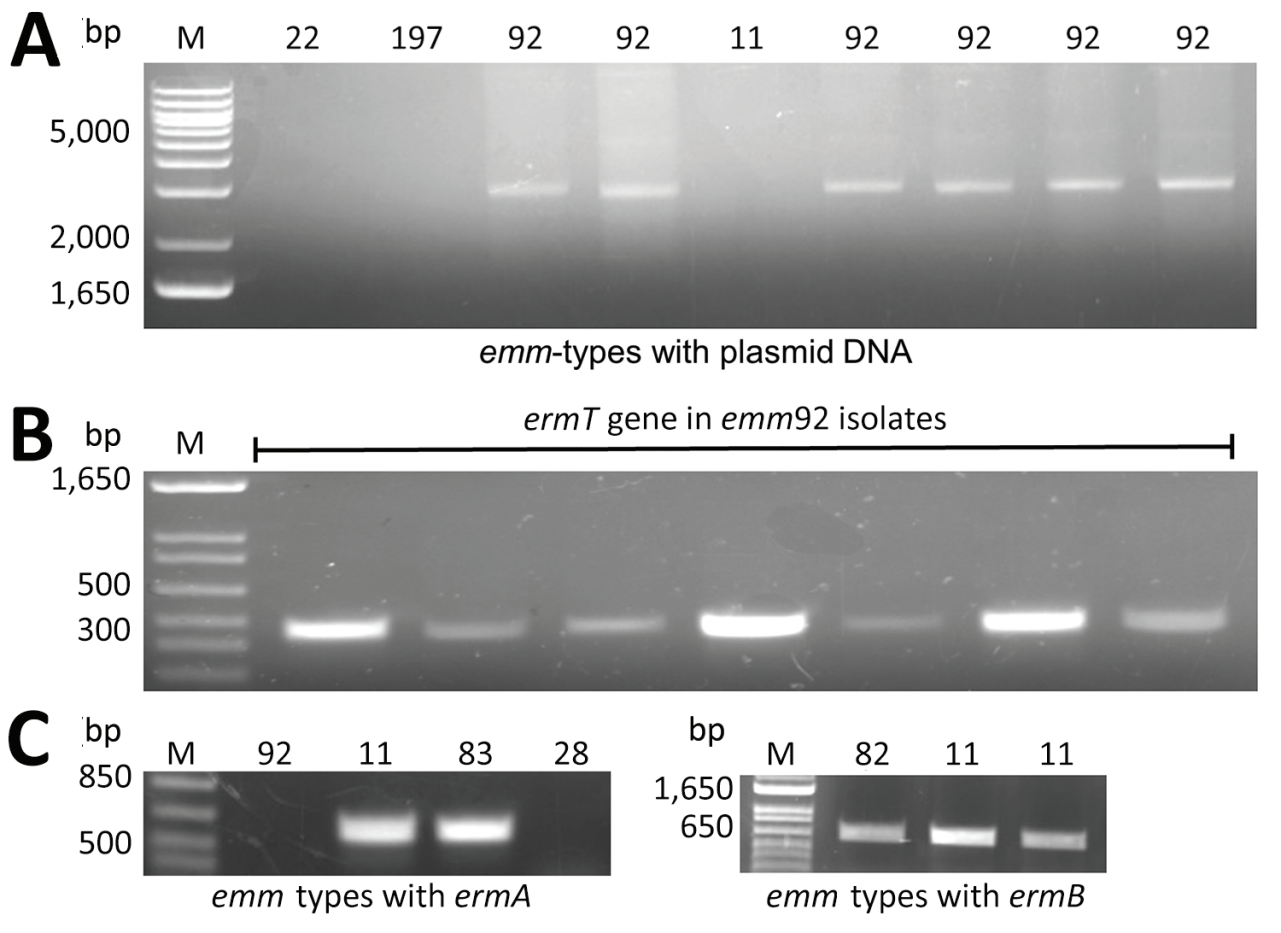
Detection of the methyl transferase genes *ermA, ermB, and ermT* in invasive group A *Streptococcus* (iGAS) isolates, West Virginia, USA, 2020–2021. A) Distribution of the pRW35-like plasmid among iGAS isolates. Presence of pRW35-like plasmid DNA was detected only in iGAS *emm92*-type isolates (representative samples are shown). B) PCR detection of the *ermT*-gene. The *ermT*-specific amplicon of 452 bp was detected in *emm92* isolates using plasmid DNA as a template. C, D) Detection of the *ermA* and *ermB* genes. Chromosomal DNA was used as a template to detect the 612-bp-*ermA* (C) and 663-bp-*ermB* (D) amplicons present in several different *emm* types. A 347-bp-*mefA* amplicon was detected in a single *emm22* isolate (data not shown).

We tested isolates for common erythromycin resistance genes by PCR amplification to detect the presence of the methyl transferase genes *ermA*, *ermB*, *and ermT*, as well as *mefA*, a gene-encoding protein associated with an efflux pump (Appendix Table 2). On the basis of a previous ABCs report that *ermT* in *emm92* GAS was carried on the pRW35 plasmid (*15*), extrachromosomal DNA was isolated from resistant isolates of various *emm* types, yet only *emm92* isolates harbored plasmid DNA (Figure 4, panel A). Restriction digestion targeting a conserved *Swa*I site confirmed a ≈4.9-kb size of this pRW35-like plasmid (data not shown). All 35 *emm92* isolates were resistant to erythromycin (Table 1) and contained *ermT* detected by PCR (Figure 4, panel B).

Chromosomal DNA was used for the detection of *ermA/B* (Figure 4, panels C, D) and *mef* (data not shown) genes. Erythromycin-resistance genes identified in *emm11* isolates varied; 6 carried *ermB* and 2 harbored *ermA* (Table 1). Of the remaining 7 erythromycin-resistant isolates of various *emm* types, *ermA* was detected in 5, *ermB* in 1, and *mefA* in 1. Collectively, 86% of *ermA-*containing isolates (6 of 7) showed inducible clindamycin resistance, whereas isolates containing *ermB* had a more evenly split phenotype for clindamycin resistance (3 iMLS_B_ vs. 4 cMLS_B_) (Table 1). The *mefA* gene, which encodes a component of the *mefA*-*msrD* efflux pump, was detected in a single *emm*22 isolate and corresponded to the erythromycin-resistant, clindamycin-susceptible phenotype referred to as the M phenotype (*20*).

### Additional Susceptibility and Proposed Resistance Determinants

Isolates also underwent susceptibility testing for tetracycline by disc diffusion, as well as for the aminoglycosides (gentamicin, streptomycin, and kanamycin) by agar dilution method using concentration ranges as previously described (*5*). In aggregate, 76% of isolates were resistant to both tetracycline and erythromycin, whereas the single *emm87* isolate was erythromycin sensitive but tetracycline resistant. For aminoglycosides, all 66 isolates were susceptible to gentamicin using CLSI *Staphylococcus aureus* breakpoints, whereas we observed presumed resistance to kanamycin (MIC >500 µg/mL) in 89% of tested isolates and resistance to streptomycin (MIC >500 µg/mL) in 61% of isolates. In addition to their universal plasmid-encoded MLS_B_ phenotype, all *emm92* strains in this collection were uniformly resistant to tetracycline, kanamycin, and streptomycin, presumably encoded by the ICESpyM92 mobile element (Table 2) (*5*). The remaining isolates of various *emm* types with MLS_B_ phenotype demonstrated resistance to either kanamycin or tetracycline.

**Table 2:** Proposed aminoglycoside and tetracycline resistance determinants in group A *Streptococcus* isolates, West Virginia, USA, 2020–2021

*emm* type	Kanamycin resistance	Streptomycin resistance	Tetracycline resistance	Determinant	Resistance element	Reference
*emm92*	+	+	+	ICESpyM92, Tn916	*Tet(M), ant (* *6* *)-Ia, aph-(3′)-III*	(*5*)
				pRW35	*ermT*	(*15*)
*emm11*	+	–	+	Tn6003-like	*Tet(M), ermB, aph(3′)-III*	(*5*)
	–	–	+	Tn6002	*Tet(M), ermB*	(*5*)
	+	–	+	ICESp2905	*Tet(O), ermA, aph-(3′)-III*	(*21*)
*emm197*	–	–	+	ICESp2905	*Tet(O), ermA*	(*21*)
*emm22*	–	–	+	Tn1207.1-like	*Tet(O), mefA,*	(*20*)

## Discussion

This study represents a comprehensive characterization of iGAS isolates from West Virginia, a geographic area beyond ABC surveillance, on the basis of the relationship between *emm* type and macrolide resistance. The results confirm a very high rate of erythromycin resistance (76%) across 7 different *emm* types producing invasive infections, most of which displayed MLS_B_ phenotype and were concomitantly resistant to clindamycin. A relationship between *emm* type and erythromycin-resistance mechanisms in this collection also became apparent. We observed that type *emm92* represented 53% of patient isolates and *emm11* 12% of patient isolates, but together those types accounted for 86% of erythromycin-resistant strains. The third most prevalent *emm* type was *emm89,* although all isolates were susceptible to erythromycin. These findings corroborate 2010–2019 nationwide data from ABCs, which demonstrated an increasing incidence of iGAS infections caused by *emm92*, *emm11*, and *emm89* types affecting the adult US population (*11*,*22*–*24*). By contrast, *emm92* iGAS infections have been reported only sporadically and at much lower frequencies globally (*25*,*26*), suggesting that expansion and dissemination of this organism might thus far be limited to the United States. Nonetheless, all 3 *emm* types are represented in the 30-valent M protein–based vaccine, signifying their role in GAS disease (*27*).

All *emm92* strains in this study harbored the pRW35-like plasmid containing the *ermT* gene, which confers resistance to erythromycin. Plasmids harboring the conserved *ermT* gene have been found in several medically relevant bacteria, including *S. pyogenes*, *S. agalactiae*, methicillin-resistant strains of *Staphylococcus aureus*, *S. gallolyticus* subspecies *pasteurianus*, and *S. suis*, which suggests horizontal gene transfer (*15*,*28*–*30*). The practice of using animal feed containing tylosin, a macrolide additive, has been suggested as a contributing factor in the spread of *ermT* across different species (*30*). The second most commonly identified *emm* type was *emm11*, in which resistance was enabled by either the *ermA* or *ermB* gene. In contrast to *emm92*, infections caused by the *emm11* strains displaying MLS_B_ phenotype have been broadly reported around the world (*4*,*31*–*34*), including fluoroquinolone-resistant isolates in China (*35*). We observed 5 iGAS infections related to *emm89.* Acapsular *emm89* strains emerged as a key cause of iGAS infections worldwide (*34*,*36*–*38*). Those strains also contain a mutation in the *nga* promoter leading to higher expression of cytotoxins NADase and streptolysin O (*36*,*39*).

We also tested resistance to tetracycline, kanamycin, streptomycin, and gentamicin. No strains were resistant to gentamicin, but all *emm92* strains demonstrated resistance to kanamycin, streptomycin, and tetracycline. Sanson et al. (*5*) used whole-genome sequencing to demonstrate that *emm92* isolates contain the ICESpyM92 element carrying the *tet(M)*, *ant (**6**)-Ia*, and *aph-(3′)-III* genes, which accounts for resistance to tetracycline and the 2 aminoglycosides (also observed in this study) and has subsequently been linked to increased virulence of *emm92* strains (*40*). Further, that research showed that *emm11* isolates harboring *ermB* and exhibiting resistance to tetracycline and kanamycin contained a Tn6003-like transposon carrying the *aph (3′)-III* gene, whereas those harboring *ermB* and tetracycline resistance alone carried Tn6002 (*5*). All 6 *emm11* isolates with *ermB* from this study displayed 1 of these 2 phenotypes. Other studies have reported ICESp2905 as the cause of resistance to tetracycline, erythromycin, and kanamycin in *emm11* isolates containing the *ermA* gene (*21*,*41*), which reflects the phenotypic pattern of 2 such isolates observed in this collection. Our collection contained 1 *emm22* isolate displaying M phenotype encoded by *mefA*, which could be carried on the transposon Tn1207.3 (*5*,*42*). Overall, these results suggest that many iGAS strains in West Virginia, especially *emm92* strains, are resistant to multiple classes of antimicrobial drugs, in addition to macrolides.

This study corroborates earlier reports noting the emergence of macrolide-resistant *emm92* iGAS nationally (*5*,*11*,*22*,*43*–*45*). We observed *emm92* as the predominant M type in every quarter, although an overall decrease in incidence was noted during July–September 2020. Quarantine related to the COVID-19 pandemic might in part explain this decrease, although periodic seasonality in disease incidence might also be a contributing factor. Nationwide data from 2010–2017 identified *emm92*, *emm49*, and *emm82* as predominant iGAS types among patients with a history of IVDU and in those experiencing homelessness (*22*), but 2020–2021 West Virginia data identified *emm92*, *emm11*, and *emm89* as the top 3 iGAS types. In addition, CDC surveillance detected considerable numbers of historically classical iGAS *emm* types (e.g., *emm1*, *emm12*, *emm28*, and *emm3)*, whereas our collection did not. Our results signify an area deserving of future investigation because of the emerging dominance of the *emm92* type in iGAS infections across the United States.

Drug abuse has become a serious epidemic in West Virginia; rates of overdose deaths have risen starting in 1999 (*46*). Patient data from this cohort corroborate that IVDU is a risk factor for resistant iGAS infections; 60.6% of affected patients reported IVDU, compared with 8.7% reported by the ABCs program. Recent studies have documented increases in drug use and overdose indicators during the COVID-19 pandemic, including higher rates of emergency department visits, emergency medical service dispatches, urine drug screen positivity, and death (*47*,*48*). Similarly, a study from Ontario, Canada, focusing on IVDU noted a 2-fold higher rate of IVDU-related SSTI than for the prepandemic era (*49*). At J.W. Ruby Memorial Hospital, overall GAS isolate recovery (calculated as total unique patient isolates per total aerobic cultures performed) declined during the pandemic, decreasing from 0.83% (890/107,150) in 2018–2019 to 0.54% (520/96,380) in 2020–2021, yet the percentage, rate, and absolute number of iGAS isolates among these totals increased substantially, from 159/892 (18%) isolates in 2018–2019 to 252/518 (49%) isolates in 2020–2021. Whether or to what extent increased IVDU during our study period affected strain diversity or resistance rates is unknown.

Homelessness among persons in West Virginia is also increasing. As of January 2020, an estimated 1,341 persons in the state experienced homelessness on any given day (*50*). In our study cohort, homelessness was reported by 13.6% of patients who had adequate documentation; 88.8% of those had a history of IVDU. In comparison, a study encompassing the 10 nationwide ABCs sites during 2010–2017 reported homelessness in 5.8% of patients and both risk factors in 6.1% of persons (*22*). 

The first limitation of our study is that, although this collection of iGAS isolates was derived from a broad geographic area of the state, it did not include all invasive strains for the periods represented. Much of southern West Virginia is beyond the WVUMed catchment area, and isolates from some WVUMed hospitals and other health systems would not have been captured. Further, because our hospital laboratory only banks invasive strains, we were unable to compare genotypic or phenotypic features of pharyngitis strains. Resistance determinants for tetracycline and aminoglycosides were not defined here. We also did not explore reasons for variable inducible/constitutive phenotype within *emm* types harboring same *erm* determinant, although research is ongoing.

In conclusion, we describe the clinicoepidemiology of iGAS infections in West Virginia over a 1.5-year period, identifying prevalent *emm* types and associated patient risk factors. Our findings indicate a particular vulnerability to iGAS infections associated with socioeconomic status, which clearly affected this study population (*46*). Further studies of *emm92* iGAS isolates and categorization of resistance will be key to improve treatments and guidelines for preventing resistance. Providing greater information and access to supplies for preventing iGAS infections in those most at risk might help reduce the spread of resistant iGAS strains in the United States. 

Appendix Additional information about the emergence of erythromycin-resistant invasive group A *Streptococcus*, West Virginia, USA, 2020–2021
