# Supplementary material for: Emergence of Erythromycin-Resistant Invasive Group A Streptococcus, West Virginia, USA, 2020–2021
Source: Emerg Infect Dis. 2023 May;29(5):898–908. doi: 10.3201/eid2905.221421 (PMC10124663; doi:10.3201/eid2905.221421)
Supplement: Appendix — Additional information about the emergence of erythromycin-resistant invasive group A Streptococcus, West Virginia, USA, 2020–2021 [file 22-1421-Techapp-s1.pdf]

Article DOI: https://doi.org/10.3201/eid2905.221421

EID cannot ensure accessibility for supplementary materials supplied by authors. Readers who have difficulty accessing supplementary content should contact the authors for assistance.

Emergence of Erythromycin-Resistant Invasive Group A *Streptococcus*, West Virginia, USA, 2020–2021

Appendix

Appendix Table 1. Isolate collection information

Patient #	*emm*- type	Age (yr)	Sex	Homeless	IVDU	Source of infection	Specimen	ICU	Surgery (No.)
1	*emm1*	71	male	no	no	Respiratory	Blood	no	no
2	*emm1*	86	female	no	no	Respiratory	Sterile fluid	yes	no
3	*emm2*	67	male	no	no	SSTI	Blood	no	no
4	*emm11*	46	female	no	yes	SSTI	Extremity	no	yes
5	*emm11*	24	female	no	yes	Deep neck	Neck aspirate	no	no
6	*emm11*	33	male	no	no	SSTI	Extremity	no	yes
7	*emm11*	41	male	no	no	SSTI	Gluteal/perirectal or inguinal	no	yes
8	*emm11*	59	male	no	no	SSTI	Extremity	no	no
9	*emm11*	50	male	no	yes	SSTI	Extremity	no	yes
10	*emm11*	43	male	yes	yes	Endovascular	Blood	yes	yes
11	*emm11*	35	male	no	yes	SSTI	Gluteal/perirectal or inguinal	no	yes
12	*emm12*	47	male	no	no	unknown	Blood	no	no
13	*emm197*	60	male	no	no	SSTI	Gluteal/perirectal or inguinal	no	no
14	*emm22*	30	female	unknown	yes	Endovascular	Blood	no	no
15	*emm28*	60	male	no	no	SSTI	Blood	yes	no
16	*emm75*	28	female	no	no	Ocular	Sterile fluid	no	no
17	*emm77*	50	male	yes	yes	unknown	Blood	no	no
18	*emm77*	32	female	no	yes	Respiratory	Sterile fluid	yes	yes
19	*emm81*	30	male	no	yes	Endovascular	Blood	no	no
20	*emm81*	34	female	yes	yes	SSTI	Extremity	yes	yes
21	*emm81*	59	male	yes	no	SSTI	Extremity	yes	yes (13)
22	*emm82*	25	male	no	yes	Endovascular	Blood	no	no
23	*emm83*	59	female	unknown	unknown	unknown	Blood	yes	no
24	*emm83*	42	female	no	yes	SSTI	Gluteal/perirectal or inguinal	no	yes
25	*emm83*	51	male	no	yes	SSTI	Extremity	no	yes (4)
26	*emm87*	36	female	no	no	Deep neck	Neck aspirate	no	no
28	*emm89*	71	female	no	no	Respiratory	Sterile fluid	yes	yes
29	*emm89*	66	male	no	no	SSTI	Extremity	no	no
30	*emm89*	46	male	no	no	SSTI	Extremity	no	yes
31	*emm89*	59	male	unknown	no	SSTI	Extremity	no	no
32	*emm92*	44	female	unknown	yes	SSTI	Extremity	no	no
34	*emm92*	48	female	yes	yes	SSTI	Extremity	no	yes (4)
37	*emm92*	62	male	no	no	SSTI	Blood	no	yes
38	*emm92*	63	male	no	no	SSTI	Blood	no	no
40	*emm92*	25	male	no	yes	Endovascular	Blood	yes	yes
41	*emm92*	26	male	no	yes	SSTI	Extremity	no	no
42	*emm92*	35	male	unknown	yes	Deep neck	Neck aspirate	no	yes
43	*emm92*	37	male	no	yes	SSTI	Extremity	no	yes

Patient #	*emm*- type	Age (yr)	Sex	Homeless	IVDU	Source of infection	Specimen	ICU	Surgery (No.)
45	*emm92*	28	male	no	yes	Deep neck	Neck aspirate	no	yes
46	*emm92*	42	male	no	yes	SSTI	Extremity	yes	yes
47	*emm92*	23	male	yes	yes	SSTI	Extremity	no	no
48	*emm92*	78	female	no	no	SSTI	Sterile fluid	yes	yes
49	*emm92*	29	male	no	yes	Endovascular	Blood	yes	yes
50	*emm92*	23	female	no	yes	SSTI	Extremity	no	yes
51	*emm92*	42	male	no	yes	SSTI	Extremity	yes	no
54	*emm92*	51	male	no	no	Deep neck	Neck aspirate	no	yes
55	*emm92*	28	female	unknown	yes	unknown	Blood	no	no
56	*emm92*	31	male	no	yes	SSTI	Extremity	no	yes
57	*emm92*	23	female	unknown	yes	SSTI	Extremity	no	no
58	*emm92*	42	female	yes	yes	SSTI	Extremity	no	no
59	*emm92*	71	female	no	no	Deep neck	Blood	yes	no
60	*emm92*	35	female	no	yes	SSTI	Extremity	no	yes
61	*emm92*	83	female	no	no	SSTI	Blood	no	no
62	*emm92*	39	male	no	yes	SSTI	Extremity	no	no
63	*emm92*	23	female	no	yes	SSTI	Extremity	no	yes
64	*emm92*	45	male	unknown	unknown	Deep neck	Neck aspirate	no	no
65	*emm92*	34	female	unknown	yes	SSTI	Extremity	no	yes
66	*emm92*	60	male	yes	yes	SSTI	Extremity	no	no
27*	*emm89*	55	male	no	no	SSTI	Blood	no	no
27*	*emm89*	55	same	same	same	same	Extremity	same	same
33*	*emm92*	33	female	no	yes	SSTI	Blood	no	yes
33*	*emm92*	same	same	same	same	same	Extremity	same	same
35*	*emm92*	47	male	no	no	SSTI	Extremity	same	yes (3)
35*	*emm92*	same	same	same	same	same	Blood	same	same
36*	*emm92*	58	female	no	no	SSTI	Extremity	yes	yes (8)
36*	*emm92*	same	same	same	same	same	Blood	same	same
39*	*emm92*	34	female	no	yes	Deep neck	Sterile fluid	yes	yes
39*	*emm92*	same	same	same	same	same	Neck aspirate	same	same
44*	*emm92*	30	female	no	yes	SSTI	Extremity	no	yes (4)
44*	*emm92*	same	same	same	same	same	Extremity	same	same
52*	*emm92*	44	male	yes	yes	SSTI	Extremity		yes
52*	*emm92*	44	same	same	same	same	same	same	same
53*	*emm92*	40	male	no	yes	Endovascular	Blood	no	no
53*	*emm92*	40	same	same	same	same	Blood	yes	yes
53*	*emm92*	same	same	same	same	same	Gluteal/perirectal or inguinal	same	same
53*	*emm92*	same	same	same	same	same	Extremity	same	same

*Replicate isolates

Appendix Table 2. Primers used for detection of *emm*-type and erythromycin (*erm*) resistance genes

Gene(s)	Primer	Sequence	Reference
emm	1b For	5′-TATTCGCTTAGAAAATTAA-3′	(*1*)
	2 Rev	5′-AAACAAGCTAAAGAACTTGC-3′	(*1*)
ermA(TR)	TR For 3	5′-ACATCTAAAAAGCATGTAAAGG-3′	(*2*)
	TR Rev 3	5′-CTTCAGCACCTGTCTTAATTG-3′	(*2*)
ermB(AM)	AM For	5′-GAAAAGGTACTCAACCAAATA-3′	(*3*)
	AM Rev	5′-AGTAACGGTACTTAAATTGTTTAC-3′	(*3*)
ermT	T For	5′-CCGCCATTGAAATAGATCCT-3′	(*4*)
	T Rev	5′- GCTTGATAAAATTGGTTTTTGGA-3′	(*4*)
mefA/E	A/E For	5′-CAGTATCATTAATCACTAGTGC-3′	(*3*)
	A/E Rev	5′-TTCTTCTGGTACTAAAAGTGG-3′	(*3*)

Appendix Table 3. Predominant *emm*-type by county

	Predominant *emm*-type per county		
Co. of Residence	*emm*-type	no. of isolates	Total no. of isolates
Allegany, MD	*emm1*	1	1
Doddridge	*emm11*	1	1
Fayette	*emm89*	1	1
Garrett, MD	*emm89*	2	3
Grant	*emm92*	1	1
Greene, PA	*emm92*	2	3
Hardy	*emm92*	1	1
Harrison	*emm92*	10	11
Jackson	*emm92*	2	2
Jefferson	*emm81*	1	1
Marion	*emm92*	6	12
Marshall	*emm81*	1	1
Mercer	*emm92*	1	1
Mineral	emm22	1	1
Monongalia	*emm92*	8	12
Nicholas	*emm92*	1	1
Non-WV/PA*	*emm92*	1	1
Pendleton	*emm11*	1	1
Preston	*emm11/89 (1 of each)*	2	2
Raleigh	*emm92*	1	1
Randolph	*emm12*	1	1
Washington, PA	*emm92*	1	1
Webster	*emm1*	1	1
Wetzel	*emm75*	1	1
Wood	*emm83*	2	3
Wyoming	*emm11*	1	1

*Isolate collected from visiting patient with a Midwestern U.S. residence

References

1. Beall B, Facklam R, Thompson T. Sequencing *emm*-specific PCR products for routine and accurate typing of group A streptococci. J Clin Microbiol. 1996;34:953–8. PubMed https://doi.org/10.1128/jcm.34.4.953-958.1996

2. Seppälä H, Skurnik M, Soini H, Roberts MC, Huovinen P. A novel erythromycin resistance methylase gene (ermTR) in Streptococcus pyogenes. Antimicrob Agents Chemother. 1998;42:257–62. PubMed https://doi.org/10.1128/AAC.42.2.257

3. Sutcliffe J, Grebe T, Tait-Kamradt A, Wondrack L. Detection of erythromycin-resistant determinants by PCR. Antimicrob Agents Chemother. 1996;40:2562–6. PubMed https://doi.org/10.1128/AAC.40.11.2562

4. Woodbury RL, Klammer KA, Xiong Y, Bailiff T, Glennen A, Bartkus JM, et al.; Active Bacterial Core Surveillance Team. Plasmid-Borne *erm(T)* from invasive, macrolide-resistant *Streptococcus pyogenes* strains. Antimicrob Agents Chemother. 2008;52:1140–3. PubMed https://doi.org/10.1128/AAC.01352-07
